# *Fusobacterium nucleatum* CbpF Mediates Inhibition of T Cell Function Through CEACAM1 Activation

**DOI:** 10.3389/fcimb.2021.692544

**Published:** 2021-07-15

**Authors:** Johanna Galaski, Amjad Shhadeh, Ariana Umaña, Christopher C. Yoo, Ludovica Arpinati, Batya Isaacson, Orit Berhani, Bernhard B. Singer, Daniel J. Slade, Gilad Bachrach, Ofer Mandelboim

**Affiliations:** ^1^ The Concern Foundation Laboratories at the Lautenberg Center for General and Tumor Immunology, Department of Immunology and Cancer Research, Institute for Medical Research Israel Canada (IMRIC), Faculty of Medicine, The Hebrew University Medical School, Jerusalem, Israel; ^2^ I. Department of Medicine, University Medical Center Hamburg-Eppendorf, Hamburg, Germany; ^3^ The Institute of Dental Sciences, The Hebrew University-Hadassah School of Dental Medicine, Jerusalem, Israel; ^4^ Department of Biochemistry, Virginia Polytechnic Institute and State University, Blacksburg, VA, United States; ^5^ Institute of Pulmonary Medicine, Hebrew University Hadassah Medical Center, Jerusalem, Israel; ^6^ Institute of Anatomy, Medical Faculty, University Duisburg-Essen, Essen, Germany

**Keywords:** *F. nucleatum*, CbpF, trimeric autotransporter adhesins, CEACAM1, CEA

## Abstract

*F. nucleatum* is an anaerobic bacterium that is associated with several tumor entities and promotes tumorigenesis. Recent evidence suggests that *F. nucleatum* binds the inhibitory receptor carcinoembryonic antigen cell adhesion molecule 1 (CEACAM1) *via* the trimeric autotransporter adhesin CbpF. However, whether this binding is functional or whether other fusobacterial trimeric autotransporter adhesins are involved in CEACAM1 activation is unknown. In this study, using *F. nucleatum* mutants lacking the type 5c trimeric autotransporter adhesins fvcA (CbpF), fvcB, fvcC, and fvcD, we show that *F. nucleatum* CbpF binds and activates CEACAM1 and also binds carcinoembryonic antigen (CEA), a tumor-associated protein. We further find that CEACAM antibodies directed against the CEACAM N-terminal domain block the CbpF-CEACAM1 interaction. In functional assays, we demonstrate CbpF-dependent inhibition of CD4^+^ T cell response. Thus, we characterize an immune evasion mechanism in which *F. nucleatum* uses its surface protein CbpF to inhibit T cell function by activating CEACAM1.

## Introduction

Tumors harbor a diverse microbiome that impacts carcinogenesis, cancer progression and therapy (Javaheri et al., 2016; Koniger et al., 2016; Gur et al., 2019a; Nejman et al., 2020). Among tumor-associated bacteria, *Fusobacterium nucleatum* has attracted increasing attention in recent years. *F. nucleatum* is a gram-negative anaerobic bacterium found in the oral cavity and associated with periodontal disease. Initially discovered to be enriched in colorectal cancer, *F. nucleatum* was since shown to be associated with esophageal (Yamamura et al., 2016), breast (Nejman et al., 2020; Parhi et al., 2020) and pancreatic cancer (Mitsuhashi et al., 2015; Nejman et al., 2020), and to promote both tumor growth and metastasis (Mima et al., 2016; Bullman et al., 2017; Parhi et al., 2020).

Besides contributing to a pro-inflammatory tumor microenvironment, *F. nucleatum* protects tumor cells from killing by NK cells and tumor infiltrating T cells. Mechanistically, we previously found that the *F. nucleatum* adhesion protein Fap2 engages TIGIT, an inhibitory receptor expressed on NK cells and T cells (Gur et al., 2015). Furthermore, we and others showed that *F. nucleatum* specifically targets carcinoembryonic antigen cell adhesion molecule 1 (CEACAM1) (Brewer et al., 2019; Gur et al., 2019b), an inhibitory receptor expressed on endothelial, epithelial, and immune cells.

CEACAM1 mediates cell adhesion *via* homophilic binding (CEACAM1-CEACAM1) or heterophilic binding to carcinoembryonic antigen (CEA), a tumor-associated adhesion molecule (Gray-Owen and Blumberg, 2006). Additionally, proteins of several bacteria have been identified as ligands for CEACAM1: *Helicobacter pylori* HopQ (Javaheri et al., 2016; Koniger et al., 2016; Gur et al., 2019a) *Neisseria* ssp. Opa proteins (Boulton and Gray-Owen, 2002), *Haemophilus influenza* P5 (Hill et al., 2001), group B *Streptococcus* β protein (van Sorge et al., 2021), *Escherichia coli* Afa/Dr adhesins (Berger et al., 2004), *Moraxella catarrhalis* UspA1 (Hill and Virji, 2003), and a yet unidentified ligand on *Acinetobacter baumanii* (Ambrosi et al., 2020). Besides bacteria, Candida albicans was found to bind CEACAM1 (Klaile et al., 2017).

Recently, the *F. nucleatum* type 5c trimeric autotransporter CbpF (CEACAM binding protein of *Fusobacterium*) was discovered to bind CEACAM1 and CEA using matrix-assisted laser desorption ionization time-of-flight (MALDI-TOF) mass spectrometry and N-terminal sequencing (Brewer et al., 2019). However, whether binding of *F. nucleatum* CbpF to CEACAM1 is functional or whether other fusobacterial trimeric autotransporter adhesins are involved in CEACAM1 activation remains unknown. In this study, using *F. nucleatum* deletion mutants of four fusobacterial 5c trimeric autotransporter proteins including CbpF, we studied the role of these proteins in CEACAM1 binding and activation.

## Materials and Methods

### Ethics

The collection of blood samples from healthy donors was approved by the Institutional Review Board of Hadassah Medical Center (HMO-0030-12).

### Primary Human T Cells and Cell Lines

To obtain primary human CD4^+^ CEACAM1-positive T cells we first isolated PBMCs from peripheral blood of healthy donors by centrifugation on Lymphoprep (StemCells Technologies). We then seeded single cells together with irradiated (6000 rad) feeder cells (50,000 allogeneic PBMCs from two donors and 5,000 RPMI 8866 cells) and 0.2 µl PHA (Sigma-Aldrich) per well in 96-well U-bottom plates. After a week, the same numbers of irradiated feeder cells were added again. Cultures were maintained in DMEM:F-12 Nutrient Mixture (70:30) supplemented with 10% human serum (Sigma Aldrich), 1% each of non-essential amino acids (Biological Industries, BI), L-glutamine (BI), sodium pyruvate (BI), and penicillin-streptomycin (BI), as well as recombinant human IL-2 (500 IU/ml, Peprotech). Following expansion, clones were stained for CD4, CD8, and CEACAM1 (Biolegend) and CEACAM1-positive CD4^+^ T cell clones were pooled together. Cells were consistently assessed for their expression of CD4, CD8, and CEACAM1 throughout the experiments.

All cell lines used in the study were originally obtained from the ATCC: human EBV-transformed 721.221 cells, mouse mastocytoma P815 cells, and mouse thymoma BW cells. Cell lines were grown in RPMI supplemented with 10% heat-inactivated fetal calf serum (FCS), 1% each of non-essential amino acids, L-glutamine, sodium pyruvate and penicillin-streptomycin (all from Biological Industries). Cells were grown at 37°C in a humidified 5% CO_2_ incubator.

### Bacteria

The generation of *F. nucleatum* ATCC 23726 gene deletion mutants is described elsewhere (Casasanta et al., 2020). All mutants were generated in the *F. nucleatum* 23726 ∆galKT background that is subsequently referred to as *Fnn*. Bacteria were grown at 37°C on anaerobic blood agar plates (Novamed) or chocolate agar plates (Novamed) under anaerobic conditions generated using Oxoid AnaeroGen 2.5L Jars and Sachets (Thermo Fisher).

### Antibodies and Fusion Proteins

The following antibodies were used: purified α-mouse IL-2 (JES6-1A12) and biotinylated α-mouse IL-2 (JES6-5H4) from Biolegend; purified α-human IFN-γ (Nib42) and biotinylated α-human IFN-γ (4S.B3) from Biolegend; purified α-human CD3 (Hit3a) from Biolegend. For flow cytometry, the following antibodies were used in blocking experiments: α-PVR (in-house antibody) used as a negative control; CEACAM antibodies 18/20 binding to the N-terminal domain of CEACAM1, 3, 5 and 6 and CC1/3/5-Sab binding to the N-terminal domain of CEACAM1, 3, 5 (B. B. Singer), C5-1X/8/8 and B3-17 binding to the A1/B1 domain of CEACAM1 (B. B. Singer), 6G5j binding to the N-terminal domain of CEACAM1, 3, 5, 6, 8 (B. B. Singer), and ASL-32 binding to CEACAM1, 5, 6 (Biolegend).

The fusion proteins CEACAM1-Fc, CEACAM3-Fc, CEACAM5-Fc, CEACAM6-Fc, CEACAM8-Fc, mouse sCEACAM1-Fc, and macaque CEACAM1-Fc were generated in HEK293T cells and purified using Protein A/G-Sepharose affinity Chromatography, as previously described (Singer et al., 2014; Javaheri et al., 2016; Moonens et al., 2018).

### FITC-Labeling of Bacteria and Flow Cytometry

Bacteria were washed twice and incubated with 0.1 mg/ml FITC (Sigma-Aldrich) in PBS at room temperature in the dark for 30 minutes. Subsequently, bacteria were washed three times in PBS to remove unbound FITC. For flow cytometry, 721.221 cells were used as carrier cells to facilitate gating. To this end, bacteria were divided into 96-well plates and incubated with 721.221 cells for 30 minutes on ice to allow for bacterial adhesion to the cells (60 million bacteria were placed together with 100,000 721.221 cells per well). Next, cells were washed and incubated with the indicated amount of CEACAM fusion proteins for 1 hour on ice followed by a 30-minute incubation with Alexa Fluor 647-conjugated donkey anti-human IgG (Jackson Immunoresearch). Histograms of cell-bound bacteria stained with CEACAM fusion proteins were gated on FITC-positive cells.

For blocking experiments, 2 µg of CEACAM1-Ig were preincubated with 1 µg of the respective antibodies for 1 hour on ice. Values obtained without blocking antibodies were arbitrarily set to 1 and all other values were normalized accordingly.

### IL-2 Release Assay From Mouse Thymoma BW Cell Transfectants

Bacteria were divided into 96 well plates (30 µl of bacteria at an OD600 of 1 per well) and incubated for one hour at 37°C in complete RPMI. Subsequently, BW CEACAM1 cells were added at 50,000 per well and incubated with the bacteria for 48 hours at 37°C. Next, supernatants were collected and mouse IL-2 levels were quantified by a sandwich ELISA. The generation of BW cells expressing chimeric CEACAM1 (composed of the extracellular portion of human CEACAM1 fused to the mouse CD3ζ chain) was previously described (Markel et al., 2002).

### Redirected Cytokine Secretion Assay

Irradiated mouse mastocytoma P815 cells that express the Fcγ receptor were coated with anti-human CD3 antibodies (25,000 P815 cells and 0.1 µg anti-CD3 per well) for 1 hour on ice. Subsequently, bacteria were added (15 million bacteria per well; 1:600). Finally, 25,000 CEACAM1-positive CD4^+^ T cells were added to each well and the plates were incubated for 48 hours at 37°C. Levels of IFN-γ in supernatants were quantified by a sandwich ELISA.

### Statistical Analysis

All statistical analyses were performed using GraphPad Prism 6. Data were statistically analyzed with one-way ANOVA followed by Dunnett’s multiple comparison test. A two-tailed unpaired *t* test was used to determine statistical significance of differences between two groups. p-values less than 0.05 were considered statistically significant (* p ≤ 0.05; ** p ≤ 0.01; *** p ≤ 0.001).

## Results

### *F. nucleatum* CbpF Binds and Activates CEACAM1

In this study, we sought to comprehensively investigate the role of fusobacterial 5c trimeric autotransporter adhesins in CEACAM1 binding. To this end, we characterized *F. nucleatum* mutants generated in the *F. nucleatum* 23726 ∆galKT strain, which allows for targeted gene deletion (Casasanta et al., 2020). In line with previously established nomenclature (Casasanta et al., 2020), we refer to this strain as *Fnn* throughout the manuscript and figures.

**Figure 1:**
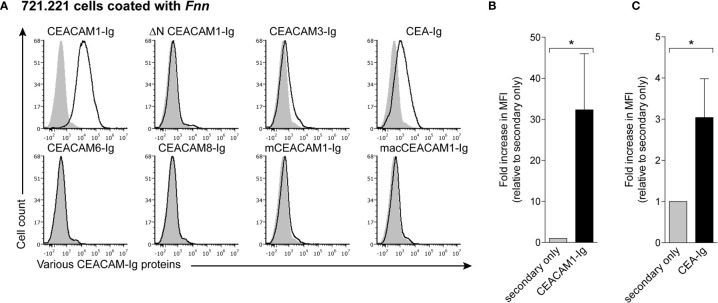
*Fnn* specifically binds to the N-terminal domain of human CEACAM1. **(A)** 721.221 cells coated with FITC-labeled *Fnn* were stained with 2 μg of human CEACAM1-Ig, ∆N CEACAM1-Ig that lacks the N-terminal domain, CEACAM3-Ig, CEA-Ig, CEACAM6-Ig, CEACAM8-Ig, mouse CEACAM1-Ig (mCEACAM1-Ig) or rhesus macaque CEACAM1-Ig (macCEACAM1-Ig). Filled grey histograms represent staining with secondary antibody only. Representative histograms from three independent repeats are shown. **(B)** CEACAM1-Ig and **(C)** CEA-Ig staining quantified and shown as fold increase in MFI relative to secondary only. Bars represent means of three independent experiments ± SD. Statistical significance was assessed using a two-tailed unpaired *t* test. *p ≤ 0.05.

To assess binding of *Fnn* to CEACAM proteins, we first stained bacteria with several different CEACAM fusion proteins in which the extracellular domain of the respective CEACAM is fused to the Fc portion of human IgG1. An overview of features of the CEACAM fusion proteins tested in this study is given in **Supplemental Table 1**. We used 721.221 cells as carrier cells for the FITC-labeled bacteria to facilitate efficient gating in flow cytometry. While 721.221 cells alone did not bind human CEACAM1-Ig, ∆N CEACAM1-Ig that lacks the N-terminal domain, CEACAM3-Ig, CEA-Ig, CEACAM6-Ig, CEACAM8-Ig, mouse CEACAM1-Ig (mCEACAM1-Ig) or rhesus macaque CEACAM1-Ig (macCEACAM1-Ig) (**Supplemental Figure 1A**), we observed staining of cell-bound *Fnn* by CEACAM1-Ig and CEA-Ig (**Figures 1A–C**), consistent with previous findings (Brewer et al., 2019; Gur et al., 2019b). In contrast, we observed no binding when bacteria were stained with ∆N-CEACAM1-Ig, indicating that the CEACAM1 N-terminal domain is critical for this interaction. Similarly, no binding of mCEACAM1-Ig and macCEACAM1-Ig homologues as well as CEACAM3-Ig, CEACAM6-Ig, and CEACAM8-Ig was detected (**Figure 1A**).

**Figure 2:**
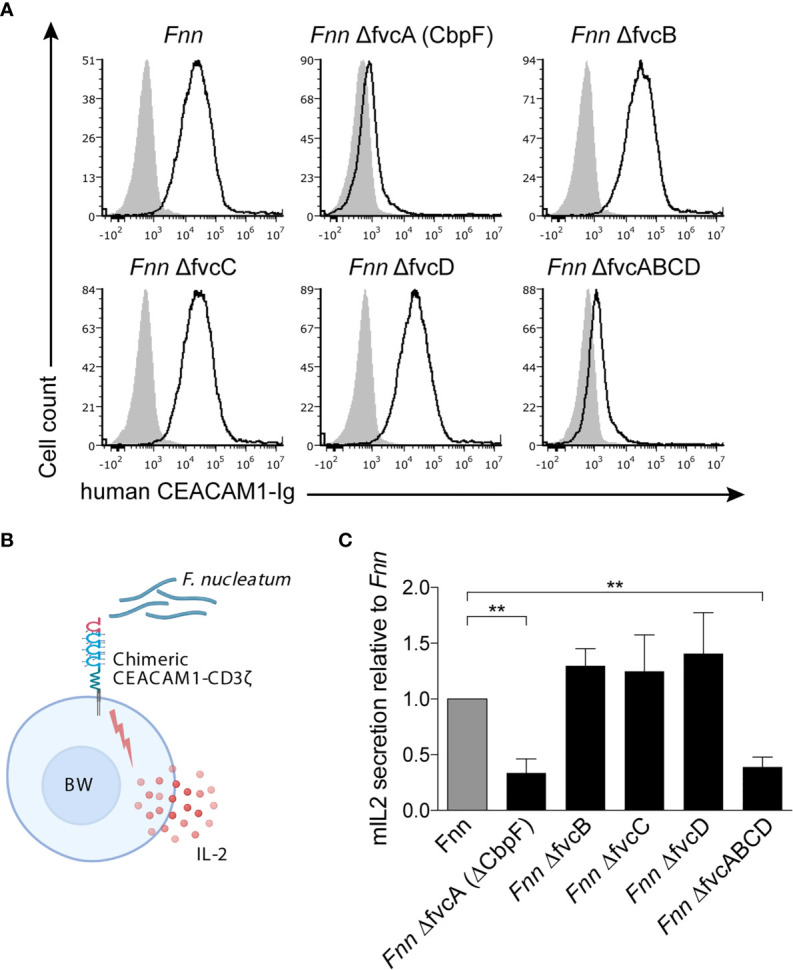
The *Fnn* ∆CbpF mutant fails to bind CEACAM1. **(A)** FITC-labeled *Fnn* and single mutants of the trimeric autotransporter adhesins fvcA (cbpF), fvcB, fvcC, fvcD, as well as a mutant lacking all four adhesins (*Fnn* fvcABCD) were stained with 3 μg of human CEACAM1-Ig. 721.221 cells were used as carrier cells. Filled gray histograms represent staining with secondary antibody only. One representative staining out of two is shown. **(B)** Schematic representation of the BW reporter assay. BW5147 cells stably transfected with human CEACAM1 fused to the transmembrane and cytoplasmic domain of mouse CD3ζ chain (chimeric recptor-CD3ζ) are incubated with above-mentioned *Fnn* strains. Activation of CEACAM1 by a ligand results in mIL-2 quantified by ELISA. **(C)** CEACAM1-expressing BW cells were incubated with *Fnn* strains at a ratio of 1:300. Mouse IL-2 in the supernatants was determined by ELISA 48 hours later. Bars represent means of three independent experiments performed in triplicates ± SD. *Fnn* bacteria were arbitrarily set to 1 and other values were normalized accordingly. Statistical significance was assessed using one-way ANOVA with Dunnett’s multiple comparison test. **p ≤ 0.01.

The trimeric autotransporter adhesin CbpF was previously suggested to be the fusobacterial ligand of CEACAM1. Therefore, we next examined binding of CEACAM1-Ig to *Fnn* single mutants each lacking one of the type 5c trimeric autotransporter adhesins fvcA (CbpF), fvcB, fvcC, fvcD, as well as a quadruple mutant deficient for all four adhesins (fvcABCD). Whereas binding of CEACAM1-Ig to the ∆fvcB, ∆fvcC and ∆fvcD mutants was similar to *Fnn*, little or no staining was observed for both *Fnn* ∆fvcA (∆CbpF) and *Fnn* ∆fvcABCD (**Figure 2A**). To corroborate these results, we used a reporter system previously generated in our lab (Markel et al., 2002). In this system, mouse thymoma BW cells expressing chimeric CEACAM1 composed of the extracellular portion of human CEACAM1 fused to the mouse CD3ζ chain secrete mouse IL-2 upon binding and activation of CEACAM1 by specific ligands (illustrated in **Figure 2B**). Whereas mouse IL-2 levels detected for *Fnn* ∆fvcB, ∆fvcC and ∆fvcD mutants were similar to *Fnn*, both the *Fnn* ∆fvcA (∆CbpF) and ∆fvcABCD mutants failed to activate CEACAM1 (**Figure 2C**). Taken together, these assays demonstrate that CbpF both binds to and activates human CEACAM1.

### The CEACAM Antibodies 18/20 and 6G5j That Target the N-Terminal Domain Block *F. nucleatum* CbpF Binding to CEACAM1

**Figure 3:**
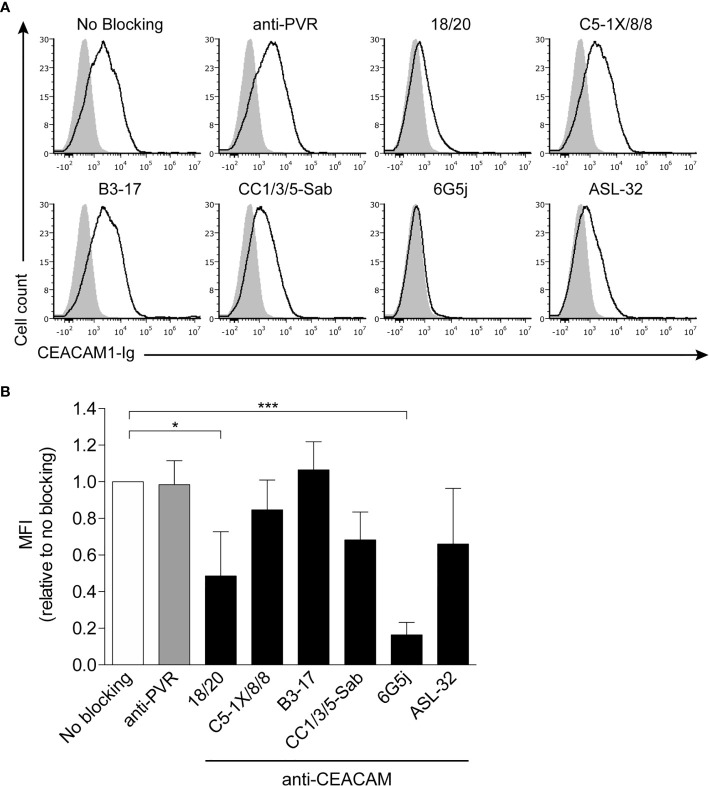
Binding of CEACAM1 to *Fnn* is blocked by anti-CEACAM1 18/20 and 6G5J. **(A)** FITC-labeled *Fnn* were stained with 2 μg of human CEACAM1-Ig preincubated with 1 µg of anti-PVR or different anti-CEACAM1 antibodies. 721.221 cells were used as carrier cells. Filled grey histograms represent staining with secondary antibody only. **(B)** Quantification of three independent experiments. Bars represent means of three independent experiments ± SD. Values obtained without blocking antibody were arbitrarily set to 1 and all other values were normalized accordingly. Statistical significance was assessed using one-way ANOVA with Dunnett’s multiple comparison test. *p ≤ 0.05; ***p ≤ 0.001.

Next, to further investigate the CEACAM1 binding site that mediates interaction with CbpF, we used different anti-CEACAM1 antibodies to attempt to block the interaction: 18/20, CC1/3/5-Sab, 6G5j, and ASL-32 that bind to the CEACAM1 N-domain; C5-1X/8/8 and B3-17 that bind to the A1-B1 domains (Javaheri et al., 2016; van Sorge et al., 2021). To assess blocking, 721.221 cell-bound FITC-labeled *Fnn* were stained with CEACAM1-Ig, following preincubation with the respective antibodies. Anti-PVR antibodies served as a negative control, since PVR is not expressed on 721.221 cells (Stanietsky et al., 2009). Both the C5-1X/8/8 and B3-17 antibodies did not significantly alter binding of CEACAM1 to CbpF, further corroborating that it is the CEACAM1 N-domain that mediates binding to CbpF (**Figure 3A**, quantification in **Figure 3B**). In contrast, blocking with 18/20 and 6G5j CEACAM antibodies led to a significant reduction in MFI (to 48.6% and 16.4%, respectively, relative to no blocking) (**Figure 3A**, quantification in **Figure 3B**). The decreases in MFI observed for CC1/3/5-Sab and ASL-32 were not statistically significant.

### CbpF Binding to CEACAM1 Leads to Inhibition of the CD4^+^ T Cell Response

**Figure 4:**
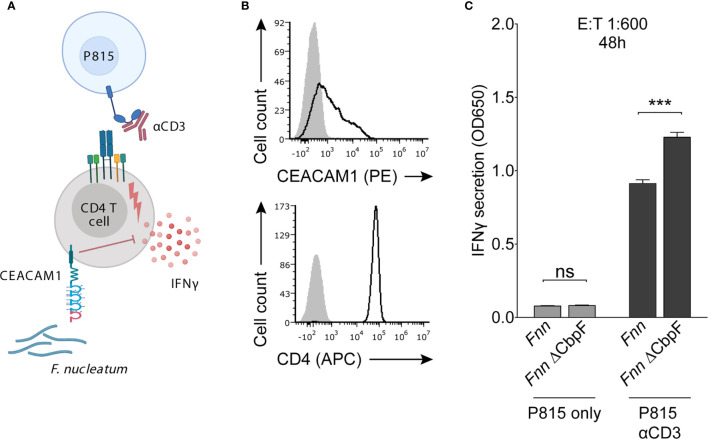
*Fnn* inhibits CD4^+^ T cell IFN-γ secretion by activating CEACAM1. **(A)** Schematic representation of the redirected cytokine assay. Mouse mastocytoma P815 cells expressing FcγR were coated with anti-CD3 to enable activation of T cells and subsequent IFN-γ secretion. Cells were incubated with *Fnn* or the *Fnn* ∆CbpF mutant at a E:T ratio of 1:600 and IFN-γ secretion was quantified by ELISA after 48 hours of incubation. **(B)** Flow cytometry staining of primary IL-2 activated human CD4^+^ T cells with anti-CEACAM1 and anti-CD4. Filled grey histograms represent staining with secondary antibody only. **(C)** Quantification of IFN-γ secretion determined by ELISA. Bars represent means of triplicates ± SD. The graphs represent data collected from two independent experiments. Statistical significance was assessed using a two-tailed unpaired *t* test. ***p ≤ 0.001. NS, not significant.

To assess the effect of CbpF on CD4^+^ T cell function, we used a redirected cytokine secretion assay. Mouse mastocytoma P815 cells expressing the Fcγ receptor were coated with an anti-CD3 antibody to enable activation of T cells (**Figure 4A**) and were further incubated with and without bacteria. The cells were then incubated with CEACAM1-positive CD4^+^ T cells that were obtained from peripheral blood of healthy donors (**Figure 4B**). The P815 cells coated with anti-CD3, CEACAM1-positive CD4^+^ T cells and *Fnn* or the *Fnn* ∆CbpF were incubated for 48 hours, and IFN-γ secretion was assessed by ELISA. In the absence of anti-CD3 antibody, little IFN-γ secretion was observed (**Figure 4C**). In contrast, in the presence of anti-CD3, IFN-γ levels were significantly higher when CEACAM1-positive CD4^+^ T cells were incubated with *Fnn* ∆CbpF as compared to *Fnn* (**Figure 4C**). These results indicate that CbpF inhibits IFN-γ secretion from CEACAM1-positive CD4^+^ T cells.

## Discussion

In this study, we used single mutants of the type 5c trimeric autotransporter adhesins fvcA (CbpF), fvcB, fvcC, and fvcD, as well as a quadruple mutant lacking all four genes, to investigate the role of these proteins in CEACAM1 binding and activation. Using both flow cytometry and functional assays, we demonstrated that CbpF binds and activates CEACAM1. The interaction was significantly blocked by the CEACAM 18/20 and 6G5j antibodies that bind the N-terminal domain. In line with these results, CEACAM1-Ig lacking the N-terminal domain failed to interact with *F. nucleatum*. Furthermore, using a redirected cytokine secretion assay, we showed that CbpF inhibits the IFN-γ response in activated CEACAM1-positive CD4^+^ T cells.

Members of the CEACAM family are known to interact with many bacteria such as *Helicobacter pylori* (Javaheri et al., 2016; Koniger et al., 2016; Gur et al., 2019a)*, Haemophilus influenza* (Hill et al., 2001), and group B *Streptoccocus* (van Sorge et al., 2021). These bacterial ligands share little structural homology (Brewer et al., 2019), strongly suggesting they evolved independently. Furthermore, not only bacteria but also Candida albicans specifically binds to CEACAM1 (Klaile et al., 2017). This notion implies a critical importance for the interaction of pathogens with CEACAM1 to allow for cellular adhesion, invasion, as well as for immune evasion.

Here we assessed the impact of CbpF-dependent CEACAM1 activation on the CD4^+^ T cell response. However, CEACAM1 is expressed not only on immune cells, but also on certain endothelial cells and epithelial cells, including cancer cells (Gray-Owen and Blumberg, 2006). The impact of *F. nucleatum* CbpF binding to CEACAM1 on these cells in the tumor microenvironment is unknown. Depending on the isoform expression profile, CEACAM1 activation can result in growth inhibition or allow for cell proliferation (Singer et al., 2000). Moreover, CEACAM1 expression on several tumor entities such as colorectal cancer (Ieda et al., 2011) and malignant melanoma (Thies et al., 2002; Thies et al., 2007) is associated with increased metastatic potential. Since *F. nucleatum* is maintained in distant metastases of CRC (Bullman et al., 2017), it will be of interest to investigate the impact of the interaction between *F. nucleatum* CbpF and CEACAM1-positive cancer cells on both cancer cell proliferation and metastasis.

## Data Availability Statement

The original contributions presented in the study are included in the article/**Supplementary Material**. Further inquiries can be directed to the corresponding author.

## Ethics Statement

The studies involving human participants were reviewed and approved by The Institutional Review Board of Hadassah Medical Center (HMO-0030-12). Written informed consent for participation was not required for this study in accordance with the national legislation and the institutional requirements.

## Author Contributions

JG and OM conceived the study. JG, AS, AU, CC, LA, BI, and OB performed the experiments. JG and OM analyzed the data. DJS contributed *F. nucleatum* mutants. BS contributed CEACAM fusion proteins and antibodies. JG drafted the manuscript. OM and GB supervised the project. All authors contributed to the article and approved the submitted version.

## Funding

JG is supported by the German Research Foundation (DFG; project number 429842436) with a postdoctoral research fellowship. This work was supported by the Israel Science Foundation (Moked grant), the GIF Foundation, the ICRF professorship grant, the ISF Israel-China grant, the MOST-DKFZ grant, and by the ERC Marie Curie grant.

## Conflict of Interest

The authors declare that the research was conducted in the absence of any commercial or financial relationships that could be construed as a potential conflict of interest.
