# Supplementary material for: Fusobacterium nucleatum CbpF Mediates Inhibition of T Cell Function Through CEACAM1 Activation
Source: Front Cell Infect Microbiol. 2021 Jul 15;11:692544. doi: 10.3389/fcimb.2021.692544 (PMC8319768; doi:10.3389/fcimb.2021.692544)
Supplement: Supplementary file 1 [file DataSheet_1.pdf]

Supplementary Material

1 Supplementary Figures and Tables

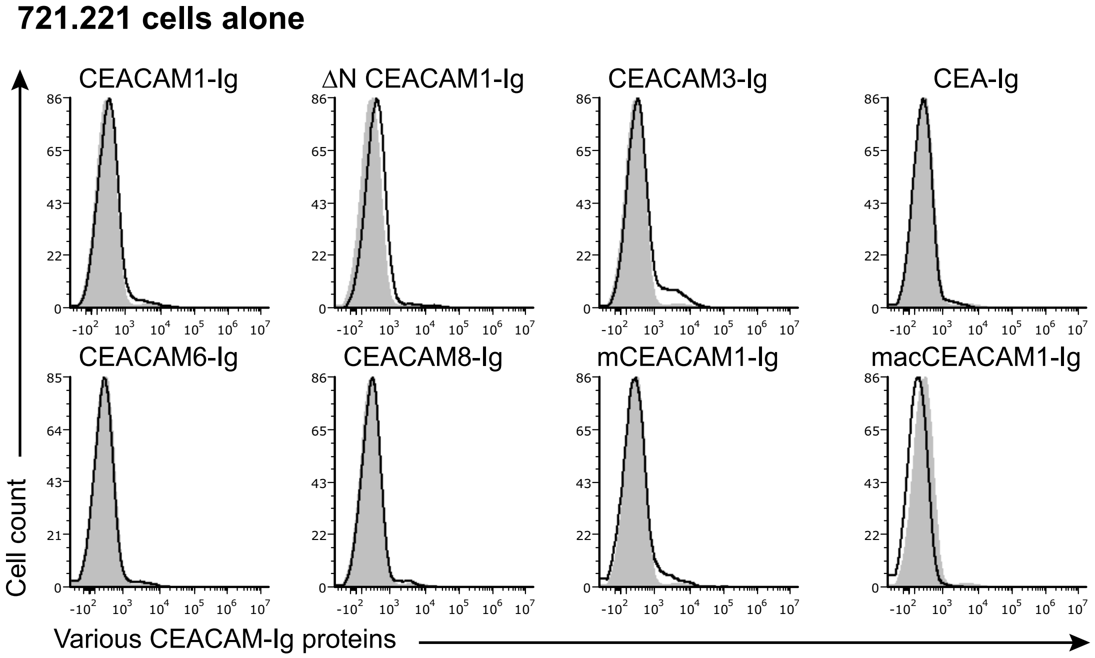

Supplemental Figure 1. Staining of 721.221 cells alone with CEACAM fusion proteins. (A) 721.221 cells alone were stained with 2 µg of human CEACAM1-Ig, ΔN CEACAM1-Ig that lacks the N-terminal domain, CEACAM3-Ig, CEA-Ig, CEACAM6-Ig, CEACAM8-Ig, mouse CEACAM1-Ig or macaque CEACAM1-Ig. Filled grey histograms represent staining with secondary antibody only. Representative histograms from three independent repeats are shown.

Supplemental Table 1. Features of CEACAM-Ig proteins used in this study

**CEACAM protein**	**Species**	**Number of extracellular domains**	**Binding to *Fnn***
CEACAM1-Ig	human	1 IgV-like domain, 3 IgC-like domains	yes
ΔN CEACAM1-Ig	human	3 IgC-like domains	no
CEACAM3-Ig	human	1 IgV-like domain	no
CEA-Ig	human	1 IgV-like domain, 6 IgC-like domains	yes
CEACAM6-Ig	human	1 IgV-like domain, 2 IgC-like domains	no
CEACAM8-Ig	human	1 IgV-like domain, 2 IgC-like domains	no
mCEACAM1-Ig	mouse	1 IgV-like domain, 3 IgC-like domains	no
macCEACAM1-Ig	macaque	1 IgV-like domain, 3 IgC-like domains	no

Fnn: Fusobacterium nucleatum 23726 ΔgalKT.
